# Standardized Preparation for Fecal Microbiota Transplantation in Pigs

**DOI:** 10.3389/fmicb.2018.01328

**Published:** 2018-06-19

**Authors:** Jun Hu, Lingli Chen, Yimei Tang, Chunlin Xie, Baoyang Xu, Min Shi, Wenyong Zheng, Shuyi Zhou, Xinkai Wang, Liu Liu, Yiqin Yan, Tao Yang, Yaorong Niu, Qiliang Hou, Xiaofan Xu, Xianghua Yan

**Affiliations:** ^1^State Key Laboratory of Agricultural Microbiology, College of Animal Sciences and Technology, Huazhong Agricultural University, Wuhan, China; ^2^The Cooperative Innovation Center for Sustainable Pig Production, Wuhan, China; ^3^Hubei Provincial Engineering Laboratory for Pig Precision Feeding and Feed Safety Technology, Wuhan, China

**Keywords:** fecal microbiota transplantation, pigs, standard, stool bank, intestinal microbiota

## Abstract

The intestine of pigs harbors a mass of microorganisms which are essential for intestinal homeostasis and host health. Intestinal microbial disorders induce enteric inflammation and metabolic dysfunction, thereby causing adverse effects on the growth and health of pigs. In the human medicine, fecal microbiota transplantation (FMT), which engrafts the fecal microbiota from a healthy donor into a patient recipient, has shown efficacy in intestinal microbiota restoration. In addition, it has been used widely in therapy for human gastrointestinal diseases, including *Clostridium difficile* infection, inflammatory bowel diseases, and irritable bowel syndrome. Given that pigs share many similarities with humans, in terms of anatomy, nutritional physiology, and intestinal microbial compositions, FMT may also be used to restore the normal intestinal microbiota of pigs. However, feasible procedures for performing FMT in pigs remains unclear. Here, we summarize a standardized preparation for FMT in pigs by combining the standard methodology for human FMT with pig production. The key issues include the donor selection, fecal material preparation, fecal material transfer, stool bank establishment, and the safety for porcine FMT. Optimal donors should be selected to ensure the efficacy of porcine FMT and reduce the risks of transmitting infectious diseases to recipients during FMT. Preparing for fresh fecal material is highly recommended. Alternatively, frozen fecal suspension can also be prepared as an optimal choice because it is convenient and has similar efficacy. Oral administration of fecal suspension could be an optimal method for porcine fecal material transfer. Furthermore, the dilution ratio of fecal materials and the frequency of fecal material transfer could be adjusted according to practical situations in the pig industry. To meet the potential large-scale requirement in the pig industry, it is important to establish a stool bank to make porcine FMT readily available. Future studies should also focus on providing more robust safety data on FMT to improve the safety and tolerability of the recipient pigs. This standardized preparation for porcine FMT can facilitate the development of microbial targeted therapies and improve the intestinal health of pigs.

## Introduction

The mammalian intestine harbors trillions of microbes (including bacteria, fungi, and viruses). These microbes play vital roles in the maintenance of gut homeostasis and host health (Sommer and Backhed, 2013). Currently, gut microbes are regarded as “microbial organs” functioning in nutrient absorption and metabolism (Backhed et al., 2007), host immune system development (Ivanov et al., 2009), the intestinal epithelium differentiation (Sommer and Backhed, 2013), and intestinal mucosal barrier maintenance (Garrett et al., 2010) in mammals. However, several factors, including host genetic characteristics, diet, environment, and antibiotic use, may affect the intestinal microbial diversity and function (Willing et al., 2011a; Schroeder and Backhed, 2016). Intestinal microbiota disorders can cause host gastrointestinal or non-gastrointestinal diseases (Brandt, 2013) such as inflammatory bowel disease (IBD), irritable bowel syndrome (IBS), and metabolic syndrome (Borody and Khoruts, 2011). Currently, antibiotics play important roles in intestinal disease prevention. However, antibiotics-induced resistance and spread of antibiotic-resistant pathogens have emerged as serious problems worldwide (Allen et al., 2014). Antibiotic therapy may also alter the intestinal microbial community and lead to intestinal microbial dysbiosis. Although probiotics (benign microbes) have shown efficacy in improving host intestinal health, their efficacy may be weak. This is because probiotic microbial composition is simple, and exogenous microbes may not colonize persistently to adapt to the dynamic intestinal homeostatic environment (Tannock et al., 2000; Sartor, 2004). Focus on the novel fecal microbiota transplantation (FMT) for the prevention and treatment of intestinal disorders has been increasing in the human medicine (Smits et al., 2013). More and more clinical applications of FMT have provided convincing proofs that modification of the intestinal microbiota is an effective therapy for intestinal dysbiosis-related diseases (Sekirov and Finlay, 2009; Smits et al., 2013). The urgent need for alternative therapies to antibiotics and the therapeutic potential of intestinal microbial manipulation promoted the development of FMT (Hamilton et al., 2012).

Fecal microbiota transplantation refers to the engraftment of fecal suspension from a healthy donor into the recipient’s intestinal tract to restore the community and function of intestinal microbiota (Khoruts and Sadowsky, 2016). The first use of donor feces as a therapeutic agent for food poisoning and diarrhea was recorded in the *Handbook of Emergency Medicine* by a Chinese, Hong Ge, in the 4^th^ century (Drew, 2016). During the 16^th^ century, Shizhen Li described the effective treatment of many intestinal diseases with fecal material in the *Compendium of Materia Medica* and the fecal suspension was called “Huanglong Tang” (Zhang et al., 2012). FMT has been applied in veterinary medicine to treat intestinal disorders of ruminants and equines since the 17^th^ century (Borody et al., 2004). In human medicine, the FMT was firstly used to treat pseudomembranous enterocolitis performed by Eiseman et al. (1958). Presently, FMT is highly recognized as an effective treatment option for recurrent *Clostridium difficile* infection (CDI) in human. It is gradually being used as a therapy for some diseases including IBD, IBS, intractable constipation, and intestinal immunodeficiency in human (van Nood et al., 2013; Borody et al., 2015). The representative cases for FMT in mammals are shown in **Table 1** (Anderson et al., 2012; Ridaura et al., 2013; van Nood et al., 2013; Sivan et al., 2015; Diao et al., 2016; Xiao et al., 2017). Growing evidences have revealed the similarity between intestinal microbiota of recipients and donors as well as the normalization of gut microbial compositions and functions in recipients after FMT therapy in human (Khoruts et al., 2010; Li et al., 2016). Rather than continuing to disturb the composition of normal intestinal microbiota, FMT efficiently restores gut microbiota of the recipients (Kelly et al., 2014). Currently, pigs encounter multiple stressors and overuse of antibiotics (Campbell et al., 2013; Barton, 2014), which destroy the normal community structure of intestinal microbiota and lead to the emergence of multidrug-resistant microorganisms in the intestine (Laxminarayan et al., 2013; Xu et al., 2015). The use of antibiotics in livestock farming was gradually banned (Casewell et al., 2003) due to that antibiotics-induced resistance, spread of antibiotic-resistant pathogens, and antibiotic residues in foods have emerged as serious problems worldwide (Allen et al., 2014). Thus, finding alternatives to antibiotics is important to livestock farming and food safety. Because of the similarities between human beings and pigs in terms of intestinal microbiota and nutritional physiology (Garthoff et al., 2002; Heinritz et al., 2013), FMT may be a promising method for intestinal microbiota reconstitution and health improvement in pigs. However, feasible procedures for performing FMT in pigs remain unclear. In this study, we summarize a standardized preparation for porcine FMT, which is used in the pig industry to prevent and treat intestinal disorders.

**Table 1:** Characteristics of donors and recipients, transplantation method, and effect of FMT on recipients.

Donors	Recipients	Transplantation method	Effect on recipients
Healthy human (Anderson et al., 2012)	Patients with IBD	Colonoscopy/enema or enteral tube	Prevent infectious diarrhea in patients with IBD
Healthy human (van Nood et al., 2013)	Patient with recurrent CDI	Colonoscopy orduodenal Infusion	Normalize bowel functioning and treat CDI
Jackson Laboratory (JAX) mice (Sivan et al., 2015)	Taconic Farms (TAC) mice	Oral gavage	Facilitate antitumor immunity
Obese twin and lean twin (Ridaura et al., 2013)	Germ-free mice	Oral gavage	Transfer the characteristics of donor obesity from human to mice
Yorkshire pigs, Tibetan pigs, and Rongchang pigs (Diao et al., 2016)	Germ-free mice	Oral gavage	Transfer the gut characteristics from pigs to mice
Yorkshire and Tibetan pigs (Xiao et al., 2017)	Commercial hybrid newborn piglets	Oral gavage	Improve the intestinal anti-inflammatory function

**Box 1. Key issues of criteria for donor screening in porcine FMT.**

**Selection of phenotypic characteristics:**

▶ Age <5 months preferably.

▶ Normal body temperature of 38∼39.5°C (rectal temperature).

▶ *Ad libitum* access to feed and water.

▶ Normal behavioral characteristics (including breathing status, feeding behavior, excreting behavior, social behavior, and reproductive behavior).

▶ No hemorrhagic spot and wound in body skin.

▶ No other abnormal behaviors.

**Risk of infectious agents for donor screening:**

▶ Recent (<2 weeks) vaccination with live attenuated virus.

▶ Recent (<2 weeks) copulation (or artificial insemination).

▶ Contact with other pigs with a history of infectious diseases in the past.

▶ Appearance of diarrhea, constipation or hematochezia.

▶ History of exposure to other endemic diarrhea areas.

▶ History of using antibiotics or other drugs.

## Donor Selection

Optimal donors should be selected to ensure the efficacy of porcine FMT and reduce the risks of transmitting infectious diseases during the transfer of fecal suspension. Selection of donors which are not fit may have adverse effects on the stability and tolerance of the intestinal microbiota, thereby causing intestinal rejection. Importantly, porcine FMT may lead to pathogen transmission because donor feces may carry pathogenic and conditional pathogenic microbes. Thus, potential donors should be selected using strict exclusion criteria, including the genetic backgrounds, phenotypic characteristics, infectious diseases, common pathogens, and other indicators. We proposed the standard for donor screening in porcine FMT based on studies related to human donors screening and pig production.

Studies have revealed that maternal-line first-degree relatives or intimate contacts (e.g., mating, common-bond) can share environmental risk factors (Owens et al., 2013). Immediate family members may contain a mass of the same microbial species in their gastrointestinal tract. As a result, recipients are more tolerant to gut microbiota from donors who are immediate family members (Kelly et al., 2015). Intestinal microbial community has been recognized to be potentially associated with the pathogenesis of diseases and intestinal disorders(Bakken et al., 2011). Phenotypic features and behaviors are the most intuitive reflections of health status in pigs. In addition, it is important to verify whether there is a history of genetic disease based on genetic spectrum analysis of ancestors of potential donors. Moreover, the potential risks of transmitting infectious diseases should be assessed. Importantly, the donor pig candidates should be isolated from other pigs to avoid the transmission of pathogens among individuals. Below are the exclusion criteria in detail (**Box 1**).

To ensure that donors are of safety for porcine FMT, serological testing and stool testing should be performed to monitor infectious pathogens and other risk factors (**Table 2**). Common infectious diseases-related pathogens in the pig industry are as follows (Meurens et al., 2012; Denner and Mueller, 2015; Lee, 2015; Renukaradhya et al., 2015; Hu et al., 2017).

**Table 2:** General serological testing and stool testing to monitor potentially infectiouspathogens.

General serological testing	Stool testing
Hog cholera virus	Porcine epidemic diarrhea virus
Pseudorabies virus	Transmissible gastroenteritis virus
Porcine parvovirus	Rotavirus
Porcine influenza virus	Bocavirus
Porcine encephalitis virus	Corona virus
Porcine reproductive and respiratory syndrome virus	pathogenic *Escherichia coli*
Porcine circovirus type 2	*Salmonella*
Cytomegalovirus	Porcine Whipworm
*Haemophilus parasuis*	Porcine ascarid
*Bordetella bronchiseptica*	Clonorchis sinensis
*Toxigenic pasteurellamultocida*	Coccidia
*Actinobacillus pleuropneumoniae*	Cysticercus cellulosae
Pathogenic *Streptococcus*	*Brachyspira hyodysenteriae*
Toxoplasma gondii	*Lawsonia intracellularis*
Porcine enterovirus	*Clostridium perfringens*
	*Shigella*

• Porcine infectious diseases-associated viruses include hog cholera virus, porcine pseudorabies virus, porcine parvovirus, porcine influenza virus, porcine encephalitis virus, porcine reproductive and respiratory syndrome virus, porcine circovirus type 2, porcine epidemic diarrhea virus, transmissible gastroenteritis virus, rotavirus, bocavirus, corona virus, cytomegalovirus, and porcine enterovirus.
• Porcine infectious diseases-associated bacteria include pathogenic *Escherichia coli*, *Salmonella*, *Haemophilus parasuis*, *Bordetella bronchiseptica*, *Toxigenic pasteurellamultocida, Actinobacillus pleuropneumoniae*, pathogenic *Streptococcus, Brachyspira hyodysenteriae, Lawsonia intracellularis*, *Clostridium perfringens*, and *Shigella*.
• Porcine infectious diseases-associated parasites include Toxoplasma gondii, porcine whipworm, porcine ascarid, Clonorchis sinensis, Coccidia, and Cysticercus cellulosae.

Serological tests are widely used to detect infectious diseases-associated pathogens based on the antigen–antibody binding reactions *in vitro*. We can use the antigen–antibody binding reactions to monitor the invasive pathogens which could stimulate host to generate the corresponding antibodies in serum. Blood samples are obtained from the porcine blood vessel and coagulated at 4°C. The serum is finally collected from the supernatant after the coagulated blood samples are centrifuged. Serological tests mainly include the serum neutralization test, hemagglutination inhibition test, enzyme-linked immunosorbent assay (ELISA), agar diffusion test, and complement fixation test. Specifically, ELISA tests have been a powerful approach to detect the infectious diseases-associated pathogens in serological tests because its several advantages, including good sensitivity and specificity (Sattler et al., 2014; Shin et al., 2015). Given that feces may carry some infectious pathogens, stool testing for donor pigs is crucial to reduce the infectious risk of porcine FMT directly. We should extract the fecal DNA from donor pigs and then perform PCR amplification reaction to confirm whether corresponding pathogens are present in the feces (Borewicz et al., 2015). RNA-virus pathogens could be detected using a combined method of the fecal RNA extraction and reverse transcription-polymerase chain reaction (RT-PCR). Specific primers should be designed according to the gene sequences of the standard pathogens. Whether the potential pathogens are present in feces could be judged through the corresponding amplified products. Moreover, ELISA test could be also used to detect pathogens-associated antigens in feces directly to confirm whether corresponding pathogens (such as porcine epidemic diarrhea virus) are present in the feces (Opriessnig et al., 2014).

Recent studies have used FMT to restore the phenotypes of donors in recipients, suggesting the key roles of intestinal microbiota in mammalian host health such as obesity (Ridaura et al., 2013; Goodrich et al., 2014), colon cancer (Wong et al., 2017), pathogens resistance (Lawley et al., 2012), and anti-tumor immunity (Sivan et al., 2015). Thus, it is crucial to select optimal pig donors because the fecal microbiota compositions of donors may have critical effects on the efficacy of the porcine FMT. Growing evidences have suggested that intestinal microbiota-mediated colonization resistance against intestinal pathogens (Buffie and Pamer, 2013). Several studies have revealed that probiotics (include *Lactobacillus*, *Bifidobacterium*, and *Bacillus* spp.) contribute to decrease the level of colonization with enterotoxigenic *E. coli* (ETEC) and maintain intestinal microbial balance (Konstantinov et al., 2008; Chiang et al., 2015). A recent study showed that *Lactobacillus johnsonii* may have the potential efficacy to reduce *Salmonella* invasion of intestinal epithelium in pigs (Casey et al., 2004). Thus, we should select optimal donor pigs which have high-abundance “functional microbes” according to the results of fecal microbial compositions and functions analyzed by 16S rDNA sequencing and metagenomics in porcine FMT.

## Preparation of Fecal Material

Previous studies have suggested that at least 30 g of fecal material should be used for the FMT in human (Mattila et al., 2012; Satokari et al., 2015). However, varieties of stool diluents, such as sterile saline (0.9%, NaCl) and phosphate buffer solution (PBS) (Gough et al., 2011; Brandt and Aroniadis, 2013) can be used as alternatives. The stool material should be diluted 3–5 times with large volumes of the solvent and buffer solution (Cammarota et al., 2017). Considering the heterogeneity in the fecal microbes between different individuals or donors (Smits et al., 2013), we suggested that the dilution ratio of the fecal materials could be adjusted in porcine FMT. Importantly, all equipment used in the fecal suspension preparation should be strictly sterile.

### Preparation of Fresh Fecal Material

**FIGURE 1:**
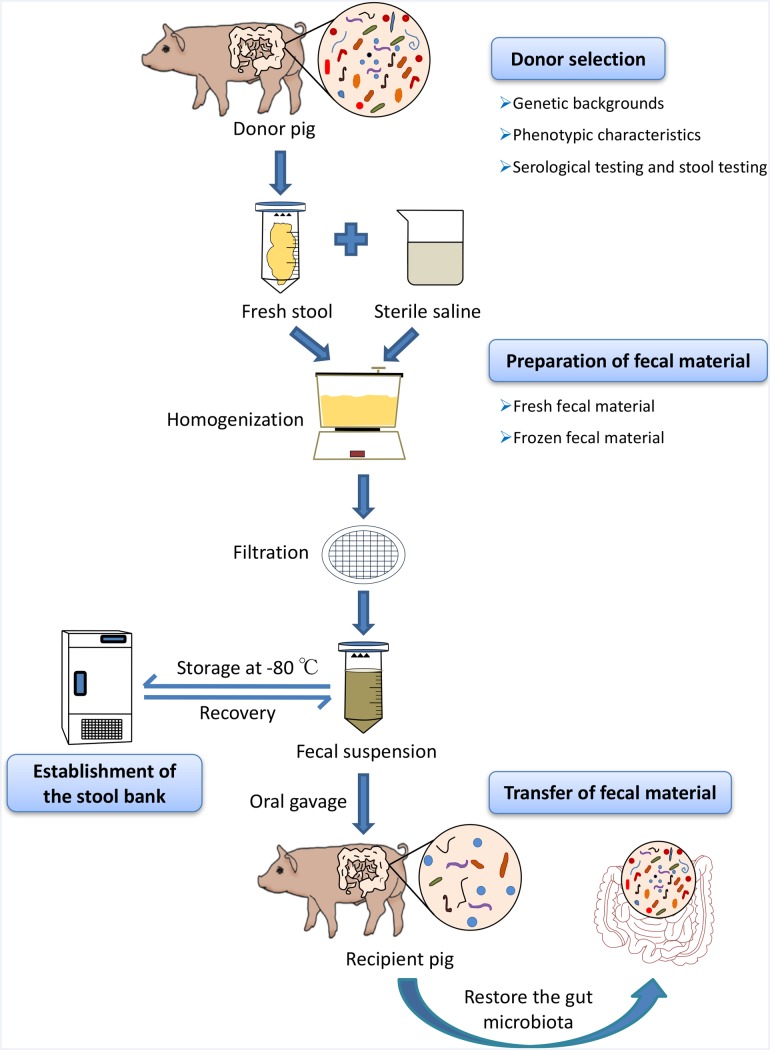
Schematic workflow of fecal microbiota transplantation (FMT) in pigs. At first, optimal donors should be selected to ensure the efficacy of porcine FMT and reduce the risks of transmitting infectious diseases during the transfer of fecal suspension via strict genetic backgrounds investigation, phenotypic characteristics, and serological test and stool testing. Fresh feces from healthy donor pigs were homogenized with sterile saline (0.9% NaCl) in blender and the stool materials should be diluted 3–5 times with large volumes of the buffer solution. The slurry was then filtered through sterile sieves and the suspension is either transferred to the recipients or mixed with 10% sterile glycerol to store at *–*80°C immediately. When there is the need for porcine FMT, the frozen fecal suspension should be thawed at 37°C (water bath).

Fresh feces used for the porcine FMT should be transported on ice to a specialized laboratory within 2 h after defecation (Lee et al., 2016). Approximately, 30 g fecal samples are diluted with 150 ml sterile saline and homogenized in a standard blender. The slurry is then filtered three times through gauze (Mattila et al., 2012), strainer, or 0.25 mm stainless steel sieves to eliminate the undigested and small particulate matter in the fecal suspension (Owens et al., 2013). We suggest that the fecal suspension could be centrifuged at 6,000 × *g* for 15 min (Hamilton et al., 2012). The precipitate, without the supernatant, is re-suspended in fresh sterile saline, and then, the resulting suspension should be transferred to the recipients directly (Hamilton et al., 2012). Because the fecal microbes are predominantly anaerobes, reducing the time of oxygen exposure in fecal material preparation is crucial to ensuring fecal microbial viability. All fecal material preparation processes should be carried out at a room temperature of 20–30°C; preferably in an anaerobic incubator (Rossen et al., 2015) (**Figure 1**).

### Preparation of Frozen Fecal Material

Preparation of frozen fecal suspension is an optimal choice to ensure sample availability, whenever there is the need for porcine FMT (Cammarota et al., 2017). Comparative studies have demonstrated that frozen fecal material does not only simplify the practical steps of clinical human FMT, but also has the similar efficacy to fresh fecal material (Satokari et al., 2015). To improve the fecal microbial survival rates during the cryopreservation, fresh stool samples should be diluted with sterile saline homogenized and filtered using the protocol used in the preparation of the fresh fecal material in porcine FMT. Subsequently, the resulting suspension should be added to glycerol to get a final concentration of 10% (Lee et al., 2016). Finally, the fecal suspensions are labeled accurately and then stored at -80°C (Satokari et al., 2015). Importantly, frozen fecal material should be stored at a low temperature as soon as possible (-80°C refrigerator or liquid nitrogen) to ensure the fecal microbial survival. When there is the need for porcine FMT, the frozen fecal suspension should be thawed at 37°C (water bath) (**Figure 1**). Upon frozen fecal suspension thawing, sterile saline solution can be added to obtain a required concentration and the infusion of fecal suspension should be implemented as soon as possible at room temperature (Satokari et al., 2015).

## Transfer of Fecal Material

In the human medicine, different routes for fecal material transfer have been reported, including the upper gastrointestinal tract (oral administration); middle gastrointestinal tract (endoscopy, nasogastric tube, nasal jejunum, and nasal duodenum); lower gastrointestinal tract (colonoscopy and enema) (Drekonja et al., 2015). In mice models, oral gavage and cohousing are used to transfer the fecal microbiota from donor mice to recipient mice (Willing et al., 2011b). Cohousing takes advantage of the natural tendency of mice to feed on the feces of littermates (Endt et al., 2010). Recent studies have suggested that porcine FMT via oral gavage using fecal suspension from donor pigs can improve growth performance, intestinal barrier, and innate immune function in recipient pigs (Hu et al., 2017; Xiao et al., 2017). However, a recent study has shown that porcine FMT via oral gavage have a negative effect on the growth performance of pigs (McCormack et al., 2018). Considering the practicality of fecal material transfer in pig production, we proposed that oral administration of fecal suspension could be an optimal method for fecal material transfer in porcine FMT (**Figure 1**). Moreover, the prepared fecal materials could be mixed with diet for direct feeding or formulated into multi-layered capsules to be administrated with diet or oral gavage directly (Hirsch et al., 2015; Youngster et al., 2016). The frequency of fecal material transfer could also be adjusted according to practical situations in pig industry. The transfer of fecal material should be performed as soon as possible because the microbial cells are fragile and sensitive.

In the human medicine, patients with CDI should be treated with vancomycin at least for 3 days and then discontinue antibiotic for 12–48 h before FMT in order to inhibit the abundance of *C. difficile* in the intestine and reduce the load of intestinal microbes (Hamilton et al., 2012; Cammarota et al., 2017). However, recent studies on FMT in animal models have shown that antibiotic pretreatment could reduce the diversity of native microbiota and may be not beneficial to the establishment of exogenous microbiota (Manichanh et al., 2010). Thus, we suggest that all recipient pigs don’t receive the antibiotics over 2 weeks before FMT and are free to water and diet.

## Establishment of the Stool Bank

To meet the potential large-scale requirement in the pig industry, it is important to establish a stool bank to make porcine FMT readily available. Fecal donors need to be recruited beforehand and rigorously screened systematically in porcine FMT. Several key issues should be considered. First, strict screening of donors including stool and serology testing is essential to prevent the transfer of infectious pathogens and reduce the risk of susceptibility in recipient (Smith et al., 2014). In addition, the supply of donor feces must meet the growing demand. Thus, we suggested that donor pigs should be segregated from other pigs since the stool and serological tests are conducted. After a series of stool and serological tests, feces will be continuously collected from eligible donor pigs (Kazerouni et al., 2015). The fecal material prepared for transplantation should be mixed with 10% sterile glycerol and stored at -80°C within 6 months, without diminishing the therapeutic efficacy (Costello et al., 2015). Establishment of stool bank not only saves the time for FMT, but also reduces cost since a single excellent donor can serve for multiple recipients (Hamilton et al., 2012). Furthermore, stool banks retain information about donors, thereby ensuring traceability during the FMT therapeutic process (Terveer et al., 2017). Procedures for the stool bank must comply with basic safety rules. Thus, the establishment of stool bank may optimize the practical procedures of porcine FMT and facilitate the development of this therapeutic method.

## Safety of FMT

Currently, most clinical experiences that focused on the use of FMT in humans have shown that FMT is safe in humans. Patients treated with FMT did not experience any serious adverse events (Borody and Khoruts, 2011), except minor symptoms such as slight diarrhea, constipation, vomiting, and abdominal discomfort (Kump et al., 2013; Rossen et al., 2015). It has been reported that the most common adverse events after FMT treatment of CDI and IBD include diarrhea, abdominal distention, abdominal cramps, constipation, and fever in human medicine (Cui et al., 2015; Agrawal et al., 2016). Some patients who received FMT treatment may suffer diarrhea on the day of transplantation, but the diarrhea generally disappears in a short term. In human medicine, adverse events are often associated with methods used to deliver fecal material, underlying diseases, and physical conditions of patients (Sokol et al., 2016). However, the evidence on the safety of FMT in pigs is relatively limited because porcine FMT has been applied before large and long-term comparable trials were conducted to assess the safety. Although recent studies have reported that the fecal microbes from donors extensively colonized in the recipients and coexisted with intestinal microbes of recipients over 3 months in human medicine (Li et al., 2016). The effects of fecal metabolites and heterogeneous substances on the intestinal microbiota of recipients are still unclear. Considering that pork is the main meat food for human, we should carefully reflect on the potential effects of porcine FMT on pork food safety. Firstly, strict donor screening is essential for reducing the risks of pathogen transmission during porcine FMT. Importantly, the potential antibiotics and drugs residues in pork of recipient pigs should be avoided via strict donor selection in which donor pigs have the history of using antibiotics or other drugs in diets or injection should not be used. Moreover, the potential effects of fecal metabolites and heterogeneous substances on pork safety should also be further investigated in porcine FMT.

## Perspectives

Based on the FMT procedures in human medicine and the pig industry, we proposed the standardized preparation (including donor selection, fecal material preparation, and fecal materials transfer) for the porcine FMT used in pig production. This standardized preparation for porcine FMT can increase the feasibility in the clinical operation for FMT and improve the intestinal health of pigs. Considering that the intestinal microbiota of piglets may be immature and sensitive to intestinal microenvironment, we conclude that porcine FMT on piglet production stage may be most effective. It is crucial to select optimal pig donors because the fecal microbes from donors may confer efficacy on the porcine FMT and the transfer of fecal materials may increase the risk of infectious pathogens transmission. Growing evidences have linked long-term diet habits to the composition of fecal microbiota (Matijasic et al., 2014). To ensure the fecal microbes from donor pigs can adapt to the intestinal microenvironment of recipient pigs, we suggest that the diets formulated according to NRC requirements for donor pigs and recipient pigs should be same if donor pigs and recipient pigs are same breed. It is known to us that there is a difference between pig breeds in diets because of the difference in nutrients requirements. Considering that there is no diet requirement for donors and recipients in human FMT, we suggest that the diets should be formulated according the nutrients requirements for different pig breeds, respectively. Although no relevant study has evaluated the survival rate of fecal microbes (including facultative anaerobic microbes, strict anaerobic microbes, and aerobic microbes) exposed to the atmosphere conditions (Yamashiro, 2017), the process of fecal material preparation will directly affect the efficacy of porcine FMT. Thus, it is important to shorten the time for fecal material preparation and transfer as soon as possible during porcine FMT. Considering that the effects of fecal metabolites and heterogeneous substances on the intestinal microbiota in recipients is still unclear, we should further improve the efficiency of fecal materials purification (besides the methods of filtration and centrifugation) to maximize the potential of porcine FMT. The method of fecal material transfer may also affect the efficacy of porcine FMT. Some microbes belonging to the phylum Firmicutes can form spores, which require growth factors in the upper digestive tract to survive (Burns et al., 2010). In addition, some microbes belonging to phylum Bacteroidetes may be denatured in the acidic environment of the stomach during the transfer (Damman et al., 2012). Therefore, it is crucial to identify the functional microbiota and choose an optimal method for delivery. A recent report has shown that the fecal microbial compositions in recipients are highly similar to that in donors by 14 days post-transplantation in human FMT (Khoruts et al., 2010). It is still difficult to conclude when the effect of FMT will be visible and how long the effect of FMT will be last because the purposes and experimental conditions for porcine FMT may be different in different assay. Thus, more studies are needed to identify the intestinal microbial dynamics induced by porcine FMT and when the effect of porcine FMT should become visible.

Fecal microbiota transplantation has been widely used in human therapy for gastrointestinal diseases, including CDI, IBD, and IBS. Interestingly, some recent studies have used FMT to restore the phenotypes of donors in recipients and suggested the key roles of intestinal microbiota in mammalian host health such as obesity (Ridaura et al., 2013; Goodrich et al., 2014), colon cancer (Wong et al., 2017), pathogens resistance (Lawley et al., 2012), and anti-tumor immunity (Sivan et al., 2015). Thus, characterization of porcine intestinal microbial functions via FMT is of great significance and requires further investigation. The underlying mechanism of FMT and the gut microbes conferring efficacy on FMT are still unclear. Thus, precise manipulation of gut microbiota through probiotics (benign microbes) has currently emerged as a promising therapeutic strategy for gastrointestinal disorders (Foxx-Orenstein and Chey, 2012; Lawley et al., 2012; Buffie et al., 2015; Schieber et al., 2015). Recently, developed high-throughput approaches (including metagenomics, metatranscriptomics, and metabolomics) have been applied to identify the association between host health and the composition and functionality of gut microbiota (Kootte et al., 2012; Costea et al., 2017). Further studies should identify specific intestinal microbial candidates that are specific to disease pathogenesis and provide novel therapeutic strategies to take advantage of such beneficial microbes (Everard et al., 2013).

## Author Contributions

JH and LC wrote the paper with the help of all authors. JH, LC, YT, CX, BX, MS, WZ, SZ, XW, LL, YY, TY, YN, QH, and XX prepared the materials for this manuscript. XY revised this manuscript. All authors read and approved the final version of the manuscript.

## Conflict of Interest Statement

The authors declare that the research was conducted in the absence of any commercial or financial relationships that could be construed as a potential conflict of interest.
